# Diagnosing Blastomycosis: A Review of Laboratory Methods and Clinical Utility

**DOI:** 10.3390/jof11080589

**Published:** 2025-08-12

**Authors:** Tejaswini Saravanababu, Sameer Elsayed, Ruchika Gupta, Johan Delport, Mohammedreza Rahimi Shahmirzadi, Fatimah AlMutawa

**Affiliations:** 1 Department of Biology, Faculty of Science, Western University, London, ON N6A 3K7, Canada; 2 Department of Infectious Diseases, Schulich School of Medicine and Dentistry, Western University, London, ON N6A 3K7, Canada; sameer.elsayed@lhsc.on.ca (S.E.); reza.rahimi@sjhc.london.on.ca (M.R.S.); 3 Department of Pathology and Laboratory Medicine, Schulich School of Medicine and Dentistry, Western University, London, ON N6A 3K7, Canada; ruchika.gupta@lhsc.on.ca (R.G.); johan.delport@lhsc.on.ca (J.D.); fatimah.almutawa@lhsc.on.ca (F.A.); 4 London Health Science Centre, London, ON N6A 5W9, Canada

**Keywords:** blastomycosis, fungal diagnostics, *Blastomyces dermatitidis*, antigen detection, PCR, serology, histopathology

## Abstract

Blastomycosis, caused by dimorphic fungi of the *Blastomyces* genus, is endemic to regions in North America, including the Great Lakes and other parts of Canada and the United States of America. The infection primarily occurs through the inhalation of airborne conidia from contaminated soil and decaying organic matter. Pulmonary involvement is most common, but dissemination to other organs such as the skin and bones can occur, especially in immunocompromised individuals. Diagnosis is challenging due to its clinical overlap with other diseases. Culture remains the gold-standard diagnostic method, but is time-consuming, with sensitivity ranging from 66.4% to 86%. Tissue histopathology offers quicker results but has sensitivities ranging from 36% to 85%. Antigen detection assays show high sensitivity from 76.3% to 91.3% but suffer from cross-reactivity with other fungi. PCR methods offer high specificity, with sensitivity ranging from 67.6% to 100%. In immunocompromised patients, blastomycosis is often more severe, with a mortality rate exceeding 30%. Multi-modal diagnostic approaches are crucial for accurate detection and management.

## 1. Introduction

Blastomycosis is a fungal infection caused by dimorphic fungi belonging to the *Blastomyces* genus, with the common species affecting humans in North America being *Blastomyces dermatitidis, Blastomyces gilchristii*, and *Blastomyces helices* [1,2]. *Blastomyces helices* has also been shown to primarily affect immunocompromised patients [3]. Other species include *Blastomyces percursus* in the Middle East and Africa, *Blastomyces emzantsi* in South Africa, *Blastomyces parvus* in Europe and Australia, and *Blastomyces silverae*, affecting animals [1]. These fungi thrive in certain environments, particularly in moist soil enriched with decaying organic matter such as wood and leaves. In North America, the infection is endemic to specific geographic regions. The highest prevalence is reported in the midwestern, south–central, and southeastern United States, with notable concentrations around the Ohio and Mississippi River valleys, the Great Lakes, and the Saint Lawrence River seaway. Northern Wisconsin and Minnesota are considered hyperendemic areas for blastomycosis [1,2,4]. Incidence rates in most regions of the United States are <1 case per 100,000 population, with hyperendemic regions in Northern Wisconsin having up to more than 20 new cases per 100,000 population [5]. Additionally, parts of Canada, including Ontario, Saskatchewan, Quebec, and Manitoba, are recognized as endemic regions with annual incidence rates ranging from around 0.113–0.62 cases per 100,000 population [6,7,8]. Northwestern Ontario is a hyperendemic region with the highest incidence rate in Canada, with 25 cases per 100,000 population [9,10]. Although less common, locally acquired cases have also been documented in Africa and India [2].

The primary route of infection is through the inhalation of airborne conidia, which are released from soil or decomposing plant material [2,11,12]. Unlike some other fungal infections, human-to-human transmission does not occur [11]. In rare cases, blastomycosis can be acquired through direct inoculation of the skin via traumatic injury [12]. The risk of infection increases with activities that disturb contaminated soil, such as construction, outdoor recreational activities and/or changes in climate, such as rainfall and temperature [9,13,14]. Certain populations, including American Indian and Alaska Natives, Asians, Native Hawaiians, and other Pacific Islanders, have been reported to have a higher incidence of blastomycosis. Additionally, immunosuppressed individuals, such as those undergoing long-term immunosuppressive therapy, organ transplant recipients, or patients with hematologic malignancies and AIDS, are at greater risk of developing severe, disseminated forms of the disease [2,13,15,16,17]

Pulmonary infection is the most common manifestation of blastomycosis, occurring in over 88–93% of cases [8,18,19,20]. Dissemination to other organs occurs in approximately 25–40% of patients [21]. The skin is the most frequent extrapulmonary site, involved in 40–80% of disseminated cases, followed by the bones in 5–25%, the genitourinary system in about 2%, and the central nervous system (CNS) in less than 2% of immunocompetent individuals [8,11,18,19,20]. However, CNS involvement occurs in 5–10% of immunocompromised individuals [22].

Given the diverse clinical manifestations of blastomycosis and significant overlap with other respiratory and systemic infections, accurate diagnosis is crucial. Various diagnostic techniques are available, but their feasibility and effectiveness are dependent on assay performance characteristics, including sensitivity, specificity, along with the patient’s immune status. This review aims to assess the diagnostic methods available for detecting blastomycosis, including culture, histopathology, antigen testing, serology, and molecular approaches, while also addressing the challenges of diagnosing immunocompromised individuals, a population that remains understudied in this context.

## 2. Specimen Handling and Safety

Proper specimen collection, transport, and handling are crucial for both accurate diagnosis and laboratory safety. Clinical samples for blastomycosis may include respiratory specimens (sputum, bronchoalveolar lavage), tissue biopsies (skin, bone, lung), blood (rarely positive), and body fluids. Urine and serum antigen tests are also used. All clinical specimens should be placed in sturdy, leak-proof containers and labeled with biohazard warnings. For shipping to reference laboratories, samples are usually classified as UN3373 (“biological substance, category B”) and packed in triple packaging per IATA/DOT guidelines (an absorbent layer inside a rigid primary container, inside a secondary container, inside an outer package) [23]. CDC guidelines recommend handling all human specimens under BSL-2 precautions by default, since the identity of the pathogen is unknown initially [24]. In practice, lab personnel wear gloves, lab coats, and eye protection when handling specimens, and any manipulation likely to generate aerosols (pipetting, vortexing, centrifugation) should be performed in a certified biosafety cabinet [24].

Special concerns arise if *Blastomyces* is suspected. Because the mold (environmental) phase produces infectious conidia, culturing clinical material on standard fungal media can generate aerosols. The CDC’s Biosafety in Microbiological and Biomedical Laboratories (BMBL) specifies that handling sporulating cultures of *B. dermatitidis* requires BSL-3 practices, equipment, and facilities [25]. Conversely, activities with yeast-phase cultures or clinical specimens (where conidia are not present) may be performed at BSL-2. In other words, processing an unknown sputum or biopsy for culture can usually be performed in a BSL-2 lab (using a Class II biosafety cabinet), but if a mold colony is grown and seen to sporulate, further work should be escalated to BSL-3 [25]. Several occupational infections with *Blastomyces* have occurred in laboratories by accidental inhalation of conidia from cultures [25]. To minimize risk, slide cultures or other methods that encourage sporulation should be avoided. Fixation and staining of smears (KOH, calcofluor) should be performed in a biosafety cabinet, and plates should not be opened outside containment. Work surfaces should be disinfected (for example, with 1–10% bleach or other sporicidal agents) after handling cultures or specimens [25].

Personnel safety also requires using personal protective equipment. In BSL-3 settings (handling mold), laboratory staff don respirators (at minimum N95 or preferably powered air-purifying respirators), solid-front gowns or coveralls, and double gloves [25]. In BSL-2 work (clinical material, yeast cultures), lab coats and gloves are standard; a surgical mask may be used if risk assessment indicates possible aerosol. All staff must be trained to recognize the risk of dimorphic fungi. Sharps precautions are critical, needle sticks can inoculate yeast forms and have caused infection in animals. Waste should be autoclaved or incinerated; spilled material should be cleaned with an appropriate disinfectant [25].

Overall, current guidelines recommend treating *Blastomyces* as a high-risk pathogen. The University of North Carolina biosafety manual (Chapter 8) explicitly notes that *B. dermatitidis* should be handled with BSL-2/BSL-3 precautions: BSL-2 for initial culture inoculation and diagnostic tests, and BSL-3 once mold forms appear [26]. BSL-3 practices, containment equipment, and facilities are recommended for handling sporulating cultures [of *B. dermatitidis*] samples containing conidia, according to BMBL [25]. These combined recommendations underscore the need for careful sample handling, adequate engineering controls, and trained staff when culturing or manipulating *Blastomyces* in the laboratory.

## 3. Diagnostic Methods

### 3.1. Culture and Histopathology

Culture remains the definitive diagnostic method for blastomycosis due to its high specificity. However, its major limitation is the extended time required for fungal growth, which can range from one to four weeks. In some cases, cultures were the first diagnostic modality to detect blastomycosis in only 3.2% of cases when compared to other diagnostic methods [27]. Clinical specimens, such as sputum, bronchoalveolar lavage (BAL), or tissue biopsies, are plated onto specialized fungal media and incubated at room temperature [28]. The growth of *Blastomyces* colonies confirms the diagnosis, but its clinical utility is diminished due to the prolonged turnaround time, with median time for the test to result in some cases being 32 days [29]. Sensitivity varies depending on the sample type, with studies reporting 86% sensitivity in same-patient samples and 75% sensitivity from a single specimen [28]. The sensitivity of culture varies depending on the type of sample plated, with bronchial secretions showing 100% sensitivity and BAL fluid at 67% [28]. Other data show culture sensitivity of 64.2–66.4% [20,30]. Despite its drawbacks, culture remains critical for confirmatory diagnosis. These data are summarized in Table 1.

Histopathological examination is an alternative diagnostic approach that offers faster results compared to culture. Histopathology, including KOH preparation and the use of special stains like PAS or GMS, can demonstrate the presence of *Blastomyces* organisms in tissue samples [31]. This method allows for a rapid presumptive diagnosis, particularly in cases where immediate clinical intervention is necessary. Histopathology is also particularly useful for detecting extrapulmonary Blastomycosis, especially in skin or bone biopsies. However, histopathology is not without limitations. Sensitivity varies from 36% for single specimens to 46% when multiple specimens are analyzed [28]. Other studies reveal sensitivities as high as 85.1% for histology and 48.4% with KOH preparation [30]. Specificities of cytology and histopathology vary by institution and evaluator, as the morphology of *Blastomyces* spp. can be confused with that of other mycoses, and need a culture to confirm species [32]. However, histopathology has been shown to offer a definitive diagnosis in certain cases [33]. The cytology of sputum and other fluids has a sensitivity range of around 93% [30,34]. Cytopathology of skin, bones, lung and other tissues shows a sensitivity of 79.5% [35]. Histopathology of bronchial brushings and bronchial lung biopsies demonstrates sensitivities of 50% and 22%, respectively [28]. An overview of these findings is presented in Table 1.

**Table 1:** Summary of the diagnostic performance of conventional methods (culture, histopathology, and serology) for the detection of *Blastomyces* species. This table outlines the sensitivity and specificity of culture from various specimen types, histopathological techniques including KOH prep and cytology, and antibody-based serologic assays such as immunodiffusion, micro-immunodiffusion, and IgG-coated well assays. Combination antibody–antigen testing is also included to demonstrate enhanced diagnostic sensitivity.

Diagnostic Method	Test Type	Sensitivity	Specificity	References
Culture	General clinical specimens	64.2–66.4%	100%	[20,28,30]
	Single vs. same-patient samples	75–86%	100%	[28]
	Bronchial secretions	100%	100%	[28]
	BAL fluid	67%	100%	[28]
Histopathology	General tissue sections	36–46%	–	[28,30,33,34,35]
	KOH prep	48.4%	–	[30]
	Cytology	93%	–	[30,33,34]
	Cytopathology	79.5%	–	[35]
	Other methods	22–50%	–	[28,30,33,34,35]
Serology (Antibody-based)	Immunodiffusion	15–64%	–	[28,36,37]
	Micro-immunodiffusion	100%	100%	[38]
	IgG-coated well assay	92.9%	79.3%	[30]
Antibody + Antigen Combo	Combined testing	97.6%	–	[36]

### 3.2. Serological Tests

Serological methods detect antibodies against *Blastomyces* antigens in patient serum. The immunodiffusion test has variable sensitivity, ranging from 15% to 40%, with moderate specificity [28,36,37]. A micro-immunodiffusion test, completed with smaller sample volumes compared to a standard immunodiffusion test, is highly accurate with 100% sensitivity and specificity [38]. One caveat of using an immunodiffusion test is that many commercial manufacturers advise that it must not be used as a standalone test, but rather in combination with histopathology or culture [39,40]. Another immunoassay utilizing microtiter wells coated with IgG anti-*B. dermatitidis* antibodies demonstrated a sensitivity of 92.9% and a specificity of 79.3% [30].

### 3.3. Antigen Detection

A second-generation urine enzyme immunoassay (EIA) from MiraVista Diagnostics is another commonly used serological test, with a sensitivity of 89.9% and specificity of 99.0%, when evaluated on a mixed infection type patient population from Marshfield Clinic in Wisconsin [41]. Blood-based antigen EIA has lower specificity due to cross-reactivity with other endemic fungi, but it remains a useful tool when used in combination with other diagnostic methods. Pulmonary disease was present in 51.7%, combined pulmonary and extrapulmonary disease in 29.2%, and extrapulmonary disease alone in 19.1% of the assessed population. Antigenuria sensitivity remains higher in patients with isolated pulmonary involvement (91.3%) or combined pulmonary and extrapulmonary involvement (92.3%) compared to isolated extrapulmonary infection (88.2%). However, there is high cross-reactivity of this immunoassay with histoplasmosis (95.6%) [41]. Antigenemia sensitivity is lower in this immunoassay at 35.7%. Sensitivity can be improved to 57.1% with EDTA pretreatment of the sample [41]. Another study demonstrates a sensitivity of antigenuria in 76.3% of patients, a sensitivity of antigenemia in 55.6%, and a positive antigen in BAL fluid in 62.5% [42]. The combination of urine and serum antigen testing yielded at least one positive result in 80% of cases, offering limited improvement in diagnostic sensitivity. Other assessments of sensitivity of the Blastomyces Antigen Quantitative EIA from MiraVista differ at 79% compared to the manufacturer’s reported sensitivity at 95.0% [43,44].

Another commercial enzyme immunoassay, namely Premier Blastomyces EIA from Meridian Diagnostics, has shown sensitivities ranging from 84 to 100% in sera and a specificity of 85.6% [38,45].

The Gotham BioTech Blastomyces (GTB) antigen urine EIA is a newer commercial test that demonstrated a 93.3% positive and 100% negative agreement in comparison with the MiraVista Diagnostics Blastomyces (MVB) EIA, while the GTB antigen serum EIA performed better with 100% positive and negative agreement with the MVB EIA [46,47,48].

An enzyme immunoassay, not commercially available, targeting the BAD-1 surface protein demonstrated a specificity of 99.0% in patients with nonfungal infections and healthy individuals; however, cross-reactivity was observed in 95.6% of patients with histoplasmosis. Combining antibody immunoassay with antigen testing increased sensitivity from 87.8% to 97.6% [49]. Sensitivities of antigen EIA tend to be higher in pulmonary manifestations compared to extrapulmonary and disseminated infections [42]. A detailed comparison of serological assays is presented in Table 1, and a comparison of antigen detection assays can be found in Table 2.

### 3.4. PCR Assay

PCR-based molecular testing offers an emerging diagnostic approach with the potential for high sensitivity and specificity. Sensitivity is estimated to be about 67.6%, which, while lower than some antigen-based methods, offers enhanced specificity compared to serological assays [37]. However, sensitivities among diagnostic methods vary. One nested PCR assay targeting the WI-1 gene showed a sensitivity of 61.5% in canine samples, with a specificity of 100% in human samples with histoplasmosis [50,51]. A TaqMan real-time PCR assay demonstrated a sensitivity of around 83%, while another newly developed real-time PCR assay demonstrated sensitivities and specificities as high as 100% from cultured isolates and 86% sensitivity and 96% specificity when performed directly on clinical specimens [52,53]. However, PCR is not yet widely standardized for clinical use, limiting its accessibility in routine diagnostics. Further research is required to refine this method and establish its role in clinical practice. The diagnostic performance of PCR-based assays is summarized in Table 3.

### 3.5. Next-Generation Sequencing

Next-generation sequencing (NGS) is an emerging diagnostic tool for blastomycosis, especially in difficult cases such as immunocompromised patients. Targeted NGS, which uses fungal-specific primers like 18S-28S rRNA gene sequencing, can detect a broad range of fungi directly from clinical samples and has successfully identified *Blastomyces dermatitidis* in lung tissue [54,55,56]. For example, ITS1 sequencing of bronchoalveolar lavage (BAL) specimens identified *Blastomyces dermatitidis/gilchristii* in 91.4% of culture-positive samples, with the fungus dominating the mycobiome (>50% relative abundance) in nearly 69% of cases, while being absent in culture-negative samples [56]. To adjust for variability in fungal biomass across samples, abundance-weighting normalization was applied, confirming significantly greater fungal loads in culture-positive specimens. This targeted approach also detected other pathogens such as Coccidioides, Scedosporium, Phaeoacremonium, and Aspergillus, highlighting its broad detection capability [56]. More studies and standardization are required before widespread application in diagnostics. Diagnostic performance of next generation sequencing summarized in Table 3.

### 3.6. MALDI-TOF MS

Matrix-assisted laser desorption/ionization time-of-flight mass spectrometry (MALDI-TOF MS) has revolutionized microbial identification by analyzing the protein spectra of cultured organisms. For *Blastomyces*, MALDI-TOF can identify the organism once a pure culture of the mold or yeast phase is obtained, using a dedicated spectral library [57]. Recent studies demonstrate that MALDI-TOF is capable of identifying dimorphic fungi. One analysis of two common MALDI-TOF systems (Bruker Biotyper and bioMérieux Vitek MS) showed that with appropriate sample preparation (often involving formic acid extraction) and updated fungal databases, isolates of *Blastomyces dermatitidis* and *B. gilchristii* can be correctly identified [57]. The test has also been shown to have 100% sensitivity and specificity [58]. Although MALDI-TOF MS offers high specificity for cultured isolates, its utility for primary clinical specimens remains limited due to biosafety level 3 (BSL-3) requirements and database gaps [58]. Diagnostic performance of MALDI-TOF MS summarized in Table 3.

**Table 3:** Molecular diagnostic methods for *Blastomyces dermatitidis* detection, including conventional PCR, TaqMan real-time PCR, and novel assays. The table compares test types, manufacturers, sensitivities, specificities, and specimen types across studies evaluating respiratory samples, cultured isolates, and Formalin-Fixed Paraffin-Embedded (FFPE) tissue biopsies.

Test Type	Manufacturer	Sensitivity	Specificity	Sample Type	Disease Type ^1^	References
PCR-Based Testing (general estimate)	Lab-developed (non-commercial)	67.6%	–	Respiratory specimens	Isolated pulmonary, Disseminated, Isolated extrapulmonary	[37]
Nested PCR (targeting WI-1 gene)	Lab-developed (non-commercial)	61.5%	100%	FFPE canine respiratory biopsy (sensitivity) FFPE human respiratory biopsy (specificity)	–	[50,51]
TaqMan Real-Time PCR	Thermo Fisher Scientific	83%	–	Culture and FFPE respiratory biopsy	–	[52]
Real-Time PCR (new assay)—cultured isolates	Lab-developed (non-commercial)	100%	100%	Fungal culture isolates	–	[53]
Real-Time PCR (new assay)—clinical specimens	Lab-developed (non-commercial)	86%	96%	Respiratory specimens	–	[53]
Next Gen Sequencing (ITS; 18S-28S primers)	Lab-developed (non-commercial)	92.2%	56%	Bronchoalveolar lavage (BAL)	–	[56]
MALDI-TOF MS	Bruker, bioMérieux	100%	100%	Fungal culture isolates	_	[57,58]

^1^ Disseminated refers to disease with both pulmonary and extrapulmonary involvement; it excludes cases with exclusive extrapulmonary involvement.

## 4. Blastomycosis in Immunocompromised Patients

Blastomycosis is increasingly recognized as a life-threatening opportunistic infection in immunocompromised individuals. The incidence of blastomycosis in immunosuppressed patients is low but significantly elevated compared to the general population, particularly among solid organ transplant (SOT) recipients. A recent retrospective study from a tertiary center in Wisconsin reported a cumulative incidence of 0.11% in SOT recipients, with infection occurring a median of 743 days post-transplant [59]. Patients with conditions that impair T-lymphocyte function, AIDS, long-term corticosteroid use, hematologic malignancies, organ transplants, or pregnancy are at heightened risk for severe and disseminated disease. Some other risk factors for Blastomycosis include pulmonary multilobar disease, obesity and other immunosuppression conditions [60]

The rate of disseminated disease was similar between groups (about 35–50%), suggesting that dissemination in blastomycosis depends more on fungal factors than host immunity; however, the rate of central nervous system involvement was higher in immunocompromised individuals [17]. Severe respiratory complications such as acute respiratory distress syndrome (ARDS) and miliary pulmonary disease are also more prevalent, with mortality rates exceeding 30% in immunocompromised individuals and up to 28% of patients dying in the first month, compared to 0 to 2% in those without immune compromise [16,17]. Beyond mortality, outcomes in immunosuppressed patients include higher rates of ICU stay, need for mechanical ventilation, and longer hospitalizations. In the Wisconsin cohort, 28% of immunocompromised patients required ICU care for acute respiratory failure, compared to 10% of immunocompetent patients [17]. Eight of the 9 immunocompromised patients with respiratory failure needed mechanical ventilation; all but one had ARDS. Among non-transplant immunosuppressed (e.g., HIV or cancer), three out of 13 were admitted to the ICU, with two requiring ventilation [17]. Relapses were uncommon overall (∼5–7%) and did not differ by immune status [17].

Immunocompromised patients have different diagnostic accuracy for blastomycosis primarily due to their reduced ability to produce antibodies, which diminishes the sensitivity of serologic tests such as enzyme immunoassays (EIA), immunodiffusion (ID), and complement fixation (CF). These assays depend on an intact humoral immune response, which may be blunted in individuals with advanced HIV infection, hematologic malignancies, solid organ transplants, or those receiving immunosuppressive medications. Consequently, these patients are at risk of false-negative results despite active infection [31]. Diagnostic manufacturers and clinical laboratories note that negative serologic results may not rule out infection in immunosuppressed individuals and recommend follow-up testing if clinical suspicion remains high [61]. Furthermore, national guidelines and infectious disease literature consistently report lower antibody detection rates in immunosuppressed patients with fungal infections, such as blastomycosis, particularly in the early stages of infection or disseminated disease [62]. These limitations underscore the need for diagnostic strategies that include antigen detection or nucleic acid-based methods in this vulnerable population.

Despite the severity of blastomycosis in immunocompromised populations, there is a significant lack of studies evaluating how the sensitivities and specificities of diagnostic tests vary in this group. While PCR-based assays may offer enhanced sensitivity, further investigation is needed to confirm their diagnostic utility in this context. Given the diagnostic challenges in immunocompromised patients, employing a combination of culture, antigen detection, and molecular testing may improve overall diagnostic accuracy [31,62].

## 5. Conclusions

Blastomycosis presents significant diagnostic challenges due to its variable clinical manifestations and symptom overlap with other systemic infections. These challenges are particularly pronounced in endemic regions, where the initial in-patient diagnosis remains markedly low, with 61% of cases not being tested until hospitalization [63]. Furthermore, diagnostic delays occur, with diagnosis being processed at a median of 31 days, particularly in immunocompromised individuals, who are at an elevated risk for severe, disseminated disease [63]. The clinical manifestations of blastomycosis are highly diverse, ranging from isolated pulmonary infection to multi-organ involvement, complicating the diagnostic process. The persistent underdiagnosis and delayed diagnosis of this infection underscore the critical need for more effective diagnostic strategies, particularly in at-risk populations.

To improve diagnostic accuracy and laboratory safety in blastomycosis, future approaches should prioritize expanding access to rapid, non-culture-based diagnostics such as lateral flow assays, antigen detection, and molecular methods, which minimize biosafety risks by avoiding fungal culture, especially in rural and resource-limited settings where the disease is endemic [2,25]. Current limitations include inconsistent sensitivity across assays, especially in immunocompromised patients, difficulty differentiating between *Blastomyces* species, and cross-reactivity with other fungi like *Histoplasma* (PubMed, PubMed). In addition, culturing *Blastomyces* poses substantial risks due to the production of infectious conidia in its mold phase, requiring BSL-3 precautions for sporulating cultures, resources that may not be universally available, leading to delayed diagnosis or underreporting [25]. Future strategies should include the development of closed-system or cartridge-based diagnostics, validated for clinical use under BSL-2 conditions, the broader implementation of standardized sample inactivation protocols that preserve nucleic acid or antigen integrity, and the expansion of laboratory biosafety training specific to dimorphic fungi [64]. Increasing surveillance, centralizing reference testing, and improving sample transport systems could also reduce diagnostic delays and improve clinical outcomes while ensuring biosafety.

Current diagnostic modalities for *Blastomyces* infection include culture, histopathology, antigen detection, serology, and molecular assays, each with distinct advantages and limitations that contribute to the diagnostic challenges encountered in clinical practice. Culture, while considered the gold standard for confirming *Blastomyces* infection, suffers from significant drawbacks, including prolonged turnaround times, exemplified by the fact that culture was the least likely method to detect Blastomycosis cases first, and variable sensitivity depending on the sample type [20,27,28,29,30]. This extended period of diagnosis allows for the progression of the infection, potentially leading to more severe disease, including systemic involvement and complications such as acute respiratory distress syndrome (ARDS) in immunocompromised patients.

Histopathological methods, including KOH preparation and special stains (e.g., PAS, GMS), offer a more rapid presumptive diagnosis compared to culture [31]. While histopathology has higher sensitivity in certain contexts (e.g., skin or bone biopsies), it remains plagued by limitations, including low specificity due to misidentification with other fungal pathogens, such as Histoplasma [28,33]. Despite these challenges, histopathology remains a valuable method, particularly in cases where expedited diagnosis is required for clinical intervention. However, its utility would be significantly enhanced by the integration of more specific diagnostic methods.

Enzyme immunoassays (EIA) for antigenuria have emerged as important tools for the diagnosis of blastomycosis, offering relatively high sensitivity in cases [38]. However, mass commercial assays have cross-reactivity with other endemic fungi, such as Histoplasma, which remains a significant concern, diminishing the specificity of these tests [41]. Combining antigen detection with other diagnostic methods, such as culture and PCR, or pretreating with EDTA, may offer a more comprehensive diagnostic approach, improving both sensitivity and specificity.

PCR assays targeting specific *Blastomyces* sequences have demonstrated sensitivity rates as high as 100% from isolates and 83% from direct specimens, with newer techniques showing even higher sensitivity and specificity [53]. Despite these promising results, the widespread clinical implementation of PCR for blastomycosis remains hindered by issues related to standardization, accessibility, and the need for specialized equipment and expertise. While PCR offers the advantage of rapid results and high specificity, it is not yet a routinely used diagnostic tool in most clinical settings.

NGS holds potential for diagnosing blastomycosis in complex cases, particularly in immunocompromised hosts. ITS1-targeted sequencing identified *Blastomyces* dermatitidis/gilchristii in 91.4% of culture-positive bronchoalveolar lavage samples, with over 50% relative abundance in 68.6% and complete absence in culture-negative specimens [56]. Despite its ability to detect a broad range of fungi, including Coccidioides and Aspergillus [54,55,56], widespread clinical use is limited by the lack of assay standardization and accessibility.

MALDI-TOF MS accurately identifies *Blastomyces* species in cultured isolates with 100% sensitivity and specificity when appropriate sample preparation and fungal databases are used [57,58]. However, current protocols require a pure culture and often BSL-3 precautions due to the infectious mold phase, restricting its role in routine diagnostics [58]. Future improvements should aim to validate workflows under BSL-2 conditions and incorporate antifungal susceptibility testing where feasible.

A critical gap in the current diagnostic framework is the lack of comprehensive studies assessing the performance of diagnostic tests in immunocompromised populations, where the disease may present in atypical or more severe forms. Immunocompromised individuals, particularly those with AIDS, organ transplants, or prolonged corticosteroid therapy, are at a higher risk for disseminated blastomycosis, often with multi-organ involvement [2,13,15,16,17]. The diagnosis of blastomycosis in these populations is further complicated by the immunosuppressive state, which may alter the host’s immune response and the pathogen’s presentation. To date, there is a lack of research specifically focusing on how various diagnostic modalities perform in immunocompromised patients. Given that mortality rates are as high as 30% with blastomycosis in these populations, targeted research is needed to evaluate and refine diagnostic approaches that can effectively identify blastomycosis early in this high-risk group [16,17].

Considering these challenges, an integrated diagnostic approach that combines multiple modalities such as antigen detection, molecular assays, culture, and histopathology offers the most promising strategy for improving diagnostic accuracy. Furthermore, efforts to standardize PCR protocols and improve accessibility to these techniques in clinical settings would significantly enhance the diagnostic landscape for blastomycosis. In conclusion, while progress has been made, substantial work remains to improve the timeliness and accuracy of blastomycosis diagnosis, particularly in vulnerable populations and endemic regions.

## Figures and Tables

**Table 2:** Diagnostic performance of enzyme immunoassays (EIA) from MiraVista Diagnostics, Meridian Bioscience, and Gotham BioTech across various sample types (urine, serum, BAL fluid). Where available, traditional sensitivity and specificity values are listed. For assays evaluated using comparative methods in the absence of a definitive gold standard, positive percent agreement (PPA) and negative percent agreement (NPA) with a reference assay (e.g., MiraVista Diagnostics’ Blastomyces antigen EIA (MVB)) are reported instead and are indicated accordingly.

Test Type	Manufacturer	Sensitivity/PPA	Specificity/NPA	Sample Type	Disease Type ^1^	References
Urine Antigen EIA	MiraVista Diagnostics	76.3–89.9%	Up to 99.0%	Urine	Isolated pulmonary, Disseminated, Isolated extrapulmonary	[41,42]
Serum Antigen EIA	MiraVista Diagnostics (Third generation)	35.7% (↑ to 57.1% with EDTA)	90% (concurrent disseminated histoplasmosis) 80% (concurrent acute histoplasmosis)	Serum	Isolated pulmonary, Disseminated, Isolated extrapulmonary	[41]
Serum Antigen EIA	MiraVista Diagnostics (Combined qualitative and quantitative EIAs)	55.6%	–	Serum	Isolated pulmonary, Disseminated	[42]
BAL Fluid Antigen EIA	MiraVista Diagnostics	62.5%	–	BAL	Isolated pulmonary, Disseminated	[42]
Blastomyces Antigen Quantitative EIA	MiraVista Diagnostics	79% (vs. 95.0% per manufacturer)	–	Urine/Serum	Isolated pulmonary, Disseminated, Isolated extrapulmonary	[43,44]
Premier Blastomyces EIA	Meridian Diagnostics	84–100%	85.6%	Serum	–	[38,45]
GTB Antigen Urine EIA	Gotham BioTech	93.3% (PPA w/MVB)	100% (NPA w/MVB)	Urine	Isolated pulmonary, Disseminated, Isolated extrapulmonary	[46,47,48]
GTB Antigen Serum EIA	Gotham BioTech	100% (PPA w/MVB)	100% (NPA w/MVB)	Serum	Isolated pulmonary, Disseminated, Isolated extrapulmonary	[46,47,48]
BAD-1 Surface Protein EIA (experimental)	Lab-developed (non-commercial)	N/A	99.2% (nonfungal), 94.0% (histoplasmosis)	Serum	Isolated pulmonary, Disseminated, Isolated extrapulmonary	[36]

^1^ Disseminated refers to disease with both pulmonary and extrapulmonary involvement; ↑ it excludes cases with exclusive extrapulmonary involvement.

## Data Availability

No new data were created or analyzed in this study. Data sharing is not applicable to this article.
